# Relationship between Vessel Diameter and Time to Maturation of Arteriovenous Fistula for Hemodialysis Access

**DOI:** 10.1155/2012/942950

**Published:** 2011-11-29

**Authors:** M. Khavanin Zadeh, F. Gholipour, Z. Naderpour, M. Porfakharan

**Affiliations:** ^1^Division of Vascular Surgery, Hasheminejad Kidney Center, Tehran University of Medical Sciences, Valinejad Street, Valiasr Avenue, Vanaq Square, Tehran 19697 14713, Iran; ^2^Tehran University of Medical Sciences, Tehran, Iran

## Abstract

*Introduction*. Native arteriovenous fistula (AVF) is the recommended vascular access for HD patients by the Dialysis Outcomes Quality Initiative (DOQI) guidelines. The aim of our study was to determine the correlation between diameter and maturation of vessels in radiocephalic AVF. *Methods. *A prospective cross-sectional study carried out during 2006-2007 on 96 hemodialysis patients from Hasheminejad Kidney Center with non probability selection, all of them with end to side native AVF*. Results*. In this population 62.5% had wrist (distal radial artery) AVF and 37.5% had antecubital (proximal radial artery) AVF. The mean diameter of artery was 2.57 mm (SD = 1.09) and the mean diameter of vein was 2.40 mm (SD = 0.79). The mean of maturation period was 38.60 days (SD = 42.13). There were no relationship between duration of maturation period and diabetes mellitus, sex, age, diameter of vein and artery (*P* > 0.05). Period of maturation showed some correlation with the diameter of vein (*P* = 0.04) in patients with distal radiocephalic fistulae. *Conclusions*. The maturation of fistula shows correlation with vein diameter, but no correlation was seen with diameter of the arteries. There is much discrepancy between times to maturation in various reports. The average time for fistula maturation was 38/6 days in our study.

## 1. Introduction

Surgery for hemodialysis (HD) access is the most commonly performed vascular surgical operation in the United States, predominantly because of a steady increase in the prevalence of end-stage renal disease (ESRD) [1]. Native arteriovenous fistula (AVF) is the recommended vascular access for HD patients by the Dialysis Outcomes Quality Initiative (DOQI) guidelines. 

The current national kidney foundation (DOQI) guidelines endorse this practice and recommend that initial cannulation be delayed for at least four weeks following surgery [2]. A fistula is considered mature when it can achieve a 300 mL/min dialysis blood flow within 1–6 months of its creation. Failure to mature (primary failure) is defined as the inability to meet this goal. 

Native AVF composed only 17% of all initial permanent hemodialysis access procedures performed in Medicare patients from 1996 to 1997 [3]. 

In 2002, the dialysis outcomes and practice patterns study (DOPPS) [2], one of the largest prospective observational studies published on hemodialysis practices and outcome in 309 international dialysis facilities, reported that AVF accounted for 24% of all access procedures in the United States, compared with 80% in Europe [4]. 

Gold standard for AVF maturation is the clinical definition of a successful maturation. It is an AVF capable of being used for successive occasions of hemodialysis.

Reported AVF maturation rate varies widely, from 30% to 90% [5–7], lower maturation rate may effectively reduce the functional patency of AVF to a level approaching that of prosthetic arteriovenous grafts [8]. 

Furthermore, AVF requires a longer period of maturation compared with prosthetic arteriovenous grafts. Protracted hemodialysis via percutaneous catheter may be required while awaiting fistula maturation, leading or increasing the risk for infection and compromise central vein patency [9]. The construction of a functional radiocephalic fistula can be challenging and high initial failure rates have been reported in many publications. Estimates of nonmaturation rated vary from just under 10% in brachiocephalic fistula to between 25% and 33% in radiocephalic fistula [10, 11]. 

The aim of our study was to determine the correlation between diameter and maturation of vessels in radiocephalic AVF. 

## 2. Methods

A prospective cross-sectional study carried out during 2006-2007 on 96 hemodialysis patients from Hasheminejad Kidney Center with nonprobability selection, all of them with end to side native AVF. 

A checklist was used for collecting data about each patient's vein and artery diameter and time of fistula maturation according to their hospital records.

Our criteria for AVF maturation was (1) easily palpable superficial vein, (2) vein relatively straight, (3) adequate diameter for easy cannulating needles (3-4 mm), (4) adequate length (≥10 cm, for adequate distance between the cannulating needles), and (5) uniform thrill to palpation and auscultation. We evaluated these criteria by nurses or nephrologists or surgeon.

Data in this study included demographic characteristics such as age, gender, and past medical history, and data about their arterial diameter and time course of maturation were collected from medical records of the enrolled patients and analyzed using the SPSS for windows software, version 16 (SPSS Inc, Chicago, Il, USA). We used descriptive and analytic tests. *P* value less than 0.05 were considered statistically significant.

## 3. Results

From the patients of Hasheminejad Kidney Center during 2006-2007, a total of 96 with native AVF with mean age 54.70 (SD = 17.17) years; 58.3% male and 41.7% female enrolled in our study. 

 In this population, 62.5% had wrist (distal radial artery) AVF, and 37.5% had antecubital (proximal radial artery) AVF. In our study, the mean diameter of artery was 2.57 mm (SD  =  1.09), and the mean diameter of vein was 2.40 mm (SD = 0.79). The mean of maturation period was 38.60 days (SD  =  42.13). 

 In antecubital AVF, proximal radial artery diameter was 3.52 mm (SD = 1.08), the mean of vein diameter was 3 mm (SD = 0.69), and maturation period in this patients was 36.05 days (SD = 36.19). In patients with distal radiocephalic fistulae, the mean of artery diameter was 2 mm (SD = 0.6), and the mean of vein diameter was 2.05 mm (SD = 0.62); in this group, the mean of maturation period of AVF was 40.13 days (SD = 45.55). 

 In our study for radiocephalic fistulae, there were no significant relationships between duration of maturation period and diabetes mellitus, sex, diameter of vein and artery, and age (*P* > 0.05). Meanwhile, in our study, period of maturation showed some correlation with the diameter of vein (*P* = 0.04) in patients with distal radiocephalic fistulae.

## 4. Discussion

The ideal hemodialysis access fistula should be durable, pose minimal risk for infection, and require few interventions to maintain ongoing functional patency. It is well documented that mature AVF demonstrate superior overall patency, lower revision rate, and cost savings, compared with prosthetic arteriovenous grafts [12]. 

 Despite almost uniform agreement on the need to increase the AVF creation rate, prevalence in the United States has increased only modestly since publication of DOQI clinical practice guideline in 1997. Less than 30% of access sites in the United States are autogenous fistula [3]. 

Cannulation, 14 days after creation was associated with 2.1-fold increased risk of subsequent fistula failure compared to fistula cannulation more than14 days. 

No significant difference in AV fistula failure was seen for fistula cannulation in 15 to 28 days compared with 43 to 48 days [13]. The finding that use of vein with larger stream diameters was associated with greater success rate was consisting with the following studies. 

 Three studies examined preoperative venous diameter and AVF adequacy for dialysis [6, 14, 15].

 Wong et al. [15] found no difference in the average venous diameter at the wrist between failed and adequate AVF but reported that all AVF failed if the diameter was 1.6 mm or less. Mendes et al. [6] reported that 16% of AVF were adequate with a vein diameter of 2 mm or less, compared to 76% of those >2 mm. 

There is much discrepancy between time of maturation in various reports. For example, the median time to first fistula cannulation differed between countries, ranging from 28 days in Japan and Italy to 96 and 98 days in transplantation in UK and US, respectively [4]. According to our data, the maturation of fistula is correlated vein diameter with, but no correlation was seen with diameter of the arteries. There is much discrepancy between time of maturation in various reports. 

 In NKF-K/DOQI guidelines, the average time for fistula maturation is reported to be 1 to 4 months, but in our study, it was 38.6 days. In some other surveys, there was a direct relation between maturation of fistula with both age and gender, but these were not seen in our study. In our data, no correlations were seen between diameter of arteries and age, gender, and diabetes. 

 Future studies should be performed with more samples to evaluate these factors and also other factors which can decreases the period of fistula maturation.
